# Supplementary material for: Multi‐dataset identification of innovative feature genes and molecular mechanisms in keratoconus
Source: J Cell Mol Med. 2024 Sep 19;28(18):e70079. doi: 10.1111/jcmm.70079 (PMC11412914; doi:10.1111/jcmm.70079)
Supplement: Supplementary file 2 — Material S2. [file JCMM-28-e70079-s002.zip › 7.GSEA analysis/3.DBNDD1/KEGG_JAK_STAT_SIGNALING_PATHWAY.html]

Details for gene set KEGG\_JAK\_STAT\_SIGNALING\_PATHWAY[GSEA]

|  || Dataset | DBNDD1.group.cls#h\_versus\_l |
| Phenotype | group.cls#h\_versus\_l |
| Upregulated in class | l |
| GeneSet | KEGG\_JAK\_STAT\_SIGNALING\_PATHWAY |
| Enrichment Score (ES) | -0.3430293 |
| Normalized Enrichment Score (NES) | -1.147524 |
| Nominal p-value | 0.27620968 |
| FDR q-value | 0.6930227 |
| FWER p-Value | 0.995 |
Table: GSEA Results Summary

  

Fig 1: Enrichment plot: KEGG\_JAK\_STAT\_SIGNALING\_PATHWAY      
 Profile of the Running ES Score & Positions of GeneSet Members on the Rank Ordered List

  

| SYMBOL | TITLE | RANK IN GENE LIST | RANK METRIC SCORE | RUNNING ES | CORE ENRICHMENT || 1 | PIK3R5 | na | 35 | 0.708 | 0.0287 | No |
| 2 | GHR | na | 396 | 0.477 | 0.0384 | No |
| 3 | IFNA14 | na | 550 | 0.438 | 0.0524 | No |
| 4 | IL21 | na | 937 | 0.390 | 0.0577 | No |
| 5 | IFNL3 | na | 1217 | 0.360 | 0.0647 | No |
| 6 | IL3RA | na | 1219 | 0.360 | 0.0798 | No |
| 7 | IL12A | na | 1227 | 0.359 | 0.0948 | No |
| 8 | IFNA6 | na | 1774 | 0.315 | 0.0922 | No |
| 9 | IL9 | na | 1908 | 0.307 | 0.1013 | No |
| 10 | IL26 | na | 2135 | 0.291 | 0.1069 | No |
| 11 | IL4R | na | 2229 | 0.286 | 0.1163 | No |
| 12 | IL7R | na | 2510 | 0.272 | 0.1196 | No |
| 13 | GH1 | na | 2516 | 0.272 | 0.1309 | No |
| 14 | IL2RB | na | 3295 | 0.239 | 0.1185 | No |
| 15 | IL3 | na | 3952 | 0.215 | 0.1085 | No |
| 16 | CBLB | na | 4045 | 0.212 | 0.1148 | No |
| 17 | IFNA16 | na | 4203 | 0.207 | 0.1189 | No |
| 18 | STAT4 | na | 5419 | 0.167 | 0.0908 | No |
| 19 | EPO | na | 5420 | 0.167 | 0.0978 | No |
| 20 | JAK3 | na | 5505 | 0.164 | 0.1023 | No |
| 21 | IL5RA | na | 6076 | 0.148 | 0.0921 | No |
| 22 | LEP | na | 6081 | 0.148 | 0.0982 | No |
| 23 | SOCS7 | na | 6529 | 0.136 | 0.0910 | No |
| 24 | IL23R | na | 6596 | 0.134 | 0.0947 | No |
| 25 | PIK3R1 | na | 6912 | 0.126 | 0.0909 | No |
| 26 | TPO | na | 7176 | 0.120 | 0.0884 | No |
| 27 | IRF9 | na | 7477 | 0.114 | 0.0845 | No |
| 28 | STAT6 | na | 7596 | 0.112 | 0.0858 | No |
| 29 | IL7 | na | 7827 | 0.107 | 0.0836 | No |
| 30 | IFNL2 | na | 7969 | 0.104 | 0.0839 | No |
| 31 | IFNE | na | 8252 | 0.099 | 0.0799 | No |
| 32 | SOS1 | na | 8586 | 0.093 | 0.0742 | No |
| 33 | CREBBP | na | 9242 | 0.082 | 0.0587 | No |
| 34 | AKT2 | na | 9388 | 0.080 | 0.0579 | No |
| 35 | CCND1 | na | 9676 | 0.076 | 0.0528 | No |
| 36 | IL2RG | na | 10291 | 0.067 | 0.0378 | No |
| 37 | IL15RA | na | 10985 | 0.058 | 0.0202 | No |
| 38 | IFNL1 | na | 11477 | 0.052 | 0.0083 | No |
| 39 | PIK3R2 | na | 11699 | 0.050 | 0.0040 | No |
| 40 | IL11RA | na | 11720 | 0.050 | 0.0055 | No |
| 41 | CBL | na | 12212 | 0.045 | -0.0068 | No |
| 42 | IL20RA | na | 12556 | 0.042 | -0.0150 | No |
| 43 | STAM2 | na | 12799 | 0.040 | -0.0203 | No |
| 44 | GH2 | na | 12931 | 0.039 | -0.0225 | No |
| 45 | IFNA2 | na | 13108 | 0.037 | -0.0260 | No |
| 46 | SOS2 | na | 13580 | 0.033 | -0.0382 | No |
| 47 | IL22 | na | 13637 | 0.033 | -0.0384 | No |
| 48 | PIK3R3 | na | 14144 | 0.029 | -0.0518 | No |
| 49 | CTF1 | na | 14147 | 0.029 | -0.0507 | No |
| 50 | CRLF2 | na | 14756 | 0.025 | -0.0672 | No |
| 51 | CSH1 | na | 14842 | 0.024 | -0.0687 | No |
| 52 | CBLC | na | 15442 | 0.020 | -0.0852 | No |
| 53 | IFNB1 | na | 15923 | 0.016 | -0.0984 | No |
| 54 | STAT5B | na | 16077 | 0.015 | -0.1022 | No |
| 55 | SOCS4 | na | 16910 | 0.010 | -0.1258 | No |
| 56 | TSLP | na | 16971 | 0.010 | -0.1271 | No |
| 57 | PIK3CA | na | 17073 | 0.009 | -0.1297 | No |
| 58 | CNTFR | na | 17407 | 0.007 | -0.1390 | No |
| 59 | SPRY1 | na | 17493 | 0.006 | -0.1412 | No |
| 60 | CCND2 | na | 17929 | 0.003 | -0.1537 | No |
| 61 | PRL | na | 18062 | 0.002 | -0.1574 | No |
| 62 | PIK3CB | na | 18338 | 0.000 | -0.1653 | No |
| 63 | PIAS1 | na | 18429 | -0.000 | -0.1679 | No |
| 64 | AKT1 | na | 19066 | -0.005 | -0.1861 | No |
| 65 | IL13RA1 | na | 19160 | -0.006 | -0.1885 | No |
| 66 | PTPN11 | na | 19596 | -0.009 | -0.2007 | No |
| 67 | IL20RB | na | 19694 | -0.010 | -0.2031 | No |
| 68 | CISH | na | 19909 | -0.012 | -0.2088 | No |
| 69 | STAT1 | na | 19966 | -0.013 | -0.2099 | No |
| 70 | STAT2 | na | 20888 | -0.021 | -0.2356 | No |
| 71 | STAM | na | 21098 | -0.023 | -0.2407 | No |
| 72 | JAK2 | na | 21143 | -0.023 | -0.2410 | No |
| 73 | SPRED1 | na | 21216 | -0.024 | -0.2421 | No |
| 74 | PIAS4 | na | 21348 | -0.025 | -0.2448 | No |
| 75 | PIAS2 | na | 21491 | -0.027 | -0.2478 | No |
| 76 | IL10RB | na | 21509 | -0.027 | -0.2471 | No |
| 77 | IFNA4 | na | 22069 | -0.033 | -0.2619 | No |
| 78 | GRB2 | na | 22294 | -0.035 | -0.2669 | No |
| 79 | CCND3 | na | 22320 | -0.035 | -0.2662 | No |
| 80 | IFNAR1 | na | 22347 | -0.036 | -0.2654 | No |
| 81 | PIAS3 | na | 22467 | -0.037 | -0.2673 | No |
| 82 | IFNGR1 | na | 22616 | -0.039 | -0.2700 | No |
| 83 | MYC | na | 22660 | -0.039 | -0.2696 | No |
| 84 | OSM | na | 23244 | -0.046 | -0.2845 | No |
| 85 | SPRY4 | na | 23559 | -0.050 | -0.2914 | No |
| 86 | IL19 | na | 23793 | -0.054 | -0.2959 | No |
| 87 | JAK1 | na | 24146 | -0.058 | -0.3037 | No |
| 88 | EP300 | na | 24292 | -0.060 | -0.3053 | No |
| 89 | PTPN6 | na | 24495 | -0.063 | -0.3085 | No |
| 90 | CSF3R | na | 24553 | -0.064 | -0.3075 | No |
| 91 | IL12RB1 | na | 24684 | -0.066 | -0.3084 | No |
| 92 | IL6 | na | 24766 | -0.067 | -0.3079 | No |
| 93 | PIK3CD | na | 24971 | -0.070 | -0.3109 | No |
| 94 | IL12B | na | 25009 | -0.071 | -0.3090 | No |
| 95 | SOCS5 | na | 25100 | -0.072 | -0.3085 | No |
| 96 | IL2RA | na | 25190 | -0.073 | -0.3080 | No |
| 97 | STAT3 | na | 25462 | -0.078 | -0.3126 | No |
| 98 | SPRY3 | na | 25761 | -0.083 | -0.3177 | No |
| 99 | IL6ST | na | 25879 | -0.085 | -0.3175 | No |
| 100 | SOCS1 | na | 25916 | -0.086 | -0.3150 | No |
| 101 | SPRY2 | na | 26563 | -0.099 | -0.3295 | No |
| 102 | BCL2L1 | na | 26569 | -0.099 | -0.3255 | No |
| 103 | STAT5A | na | 26873 | -0.105 | -0.3298 | No |
| 104 | CSF2RB | na | 26951 | -0.107 | -0.3276 | No |
| 105 | PIK3CG | na | 27455 | -0.118 | -0.3372 | No |
| 106 | IFNGR2 | na | 27570 | -0.120 | -0.3354 | No |
| 107 | TYK2 | na | 27835 | -0.127 | -0.3377 | Yes |
| 108 | IL23A | na | 27929 | -0.129 | -0.3349 | Yes |
| 109 | MPL | na | 28023 | -0.132 | -0.3321 | Yes |
| 110 | IL13 | na | 28093 | -0.133 | -0.3285 | Yes |
| 111 | IL12RB2 | na | 28204 | -0.135 | -0.3260 | Yes |
| 112 | IFNAR2 | na | 28242 | -0.136 | -0.3213 | Yes |
| 113 | IL6R | na | 28280 | -0.137 | -0.3166 | Yes |
| 114 | IL5 | na | 28795 | -0.151 | -0.3251 | Yes |
| 115 | LEPR | na | 29224 | -0.163 | -0.3306 | Yes |
| 116 | LIFR | na | 29236 | -0.164 | -0.3240 | Yes |
| 117 | IL2 | na | 29432 | -0.170 | -0.3225 | Yes |
| 118 | PIM1 | na | 29455 | -0.171 | -0.3160 | Yes |
| 119 | IL9R | na | 29538 | -0.174 | -0.3111 | Yes |
| 120 | SPRED2 | na | 29640 | -0.177 | -0.3066 | Yes |
| 121 | CSF3 | na | 30125 | -0.192 | -0.3125 | Yes |
| 122 | CLCF1 | na | 30127 | -0.192 | -0.3044 | Yes |
| 123 | CSF2 | na | 30210 | -0.195 | -0.2986 | Yes |
| 124 | IFNA8 | na | 30493 | -0.203 | -0.2982 | Yes |
| 125 | IL20 | na | 30671 | -0.210 | -0.2945 | Yes |
| 126 | IFNA7 | na | 30811 | -0.214 | -0.2895 | Yes |
| 127 | IL15 | na | 31024 | -0.221 | -0.2863 | Yes |
| 128 | EPOR | na | 31178 | -0.227 | -0.2812 | Yes |
| 129 | IL10 | na | 31214 | -0.229 | -0.2726 | Yes |
| 130 | IL22RA1 | na | 31485 | -0.239 | -0.2703 | Yes |
| 131 | SOCS3 | na | 31574 | -0.244 | -0.2626 | Yes |
| 132 | IFNW1 | na | 31807 | -0.255 | -0.2586 | Yes |
| 133 | LIF | na | 32371 | -0.286 | -0.2629 | Yes |
| 134 | IL21R | na | 32838 | -0.316 | -0.2631 | Yes |
| 135 | IFNA10 | na | 32985 | -0.328 | -0.2535 | Yes |
| 136 | CNTF | na | 33174 | -0.342 | -0.2445 | Yes |
| 137 | CSF2RA | na | 33430 | -0.364 | -0.2366 | Yes |
| 138 | PRLR | na | 33449 | -0.366 | -0.2217 | Yes |
| 139 | IFNLR1 | na | 33526 | -0.372 | -0.2083 | Yes |
| 140 | IL24 | na | 33613 | -0.381 | -0.1948 | Yes |
| 141 | AKT3 | na | 33656 | -0.385 | -0.1798 | Yes |
| 142 | IL4 | na | 33666 | -0.386 | -0.1638 | Yes |
| 143 | IFNG | na | 33717 | -0.393 | -0.1487 | Yes |
| 144 | IFNA21 | na | 34135 | -0.449 | -0.1419 | Yes |
| 145 | IFNK | na | 34142 | -0.450 | -0.1232 | Yes |
| 146 | IL13RA2 | na | 34220 | -0.468 | -0.1058 | Yes |
| 147 | IL11 | na | 34460 | -0.537 | -0.0901 | Yes |
| 148 | OSMR | na | 34470 | -0.540 | -0.0676 | Yes |
| 149 | IL10RA | na | 34529 | -0.565 | -0.0456 | Yes |
| 150 | IL22RA2 | na | 34572 | -0.598 | -0.0216 | Yes |
| 151 | SOCS2 | na | 34593 | -0.621 | 0.0039 | Yes |
Table: GSEA details [plain text format]

  

Fig 2: KEGG\_JAK\_STAT\_SIGNALING\_PATHWAY      
 Blue-Pink O' Gram in the Space of the Analyzed GeneSet

  

Fig 3: KEGG\_JAK\_STAT\_SIGNALING\_PATHWAY: Random ES distribution      
 Gene set null distribution of ES for **KEGG\_JAK\_STAT\_SIGNALING\_PATHWAY**
